# Supplementary material for: Blockade of leukemia inhibitory factor as a therapeutic approach to KRAS driven pancreatic cancer
Source: Nat Commun. 2019 Jul 11;10:3055. doi: 10.1038/s41467-019-11044-9 (PMC6624260; doi:10.1038/s41467-019-11044-9)
Supplement: Supplementary file 1 — Supplementary Information [file 41467_2019_11044_MOESM1_ESM.pdf]

Supplementary Figure 1

a

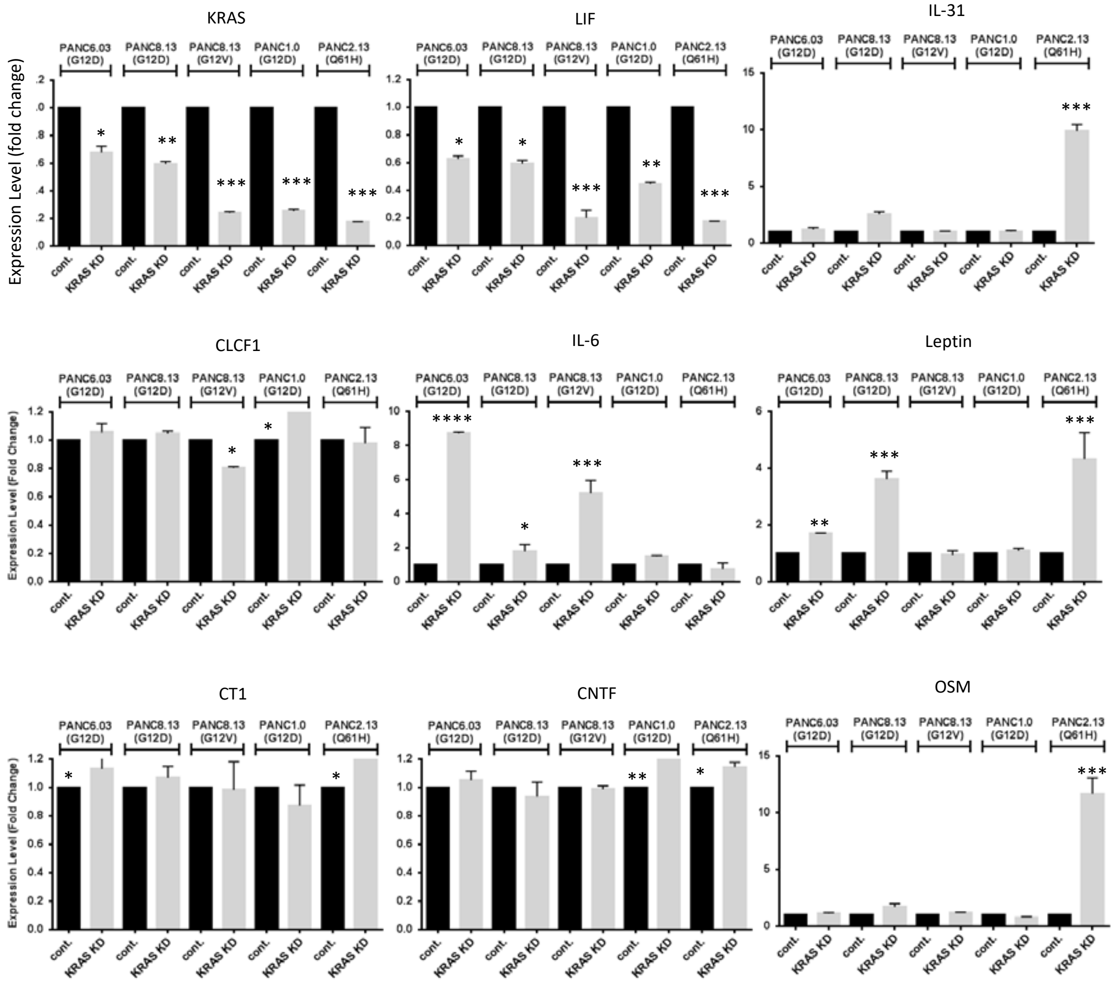

b

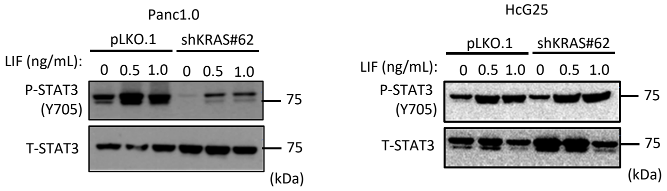

c

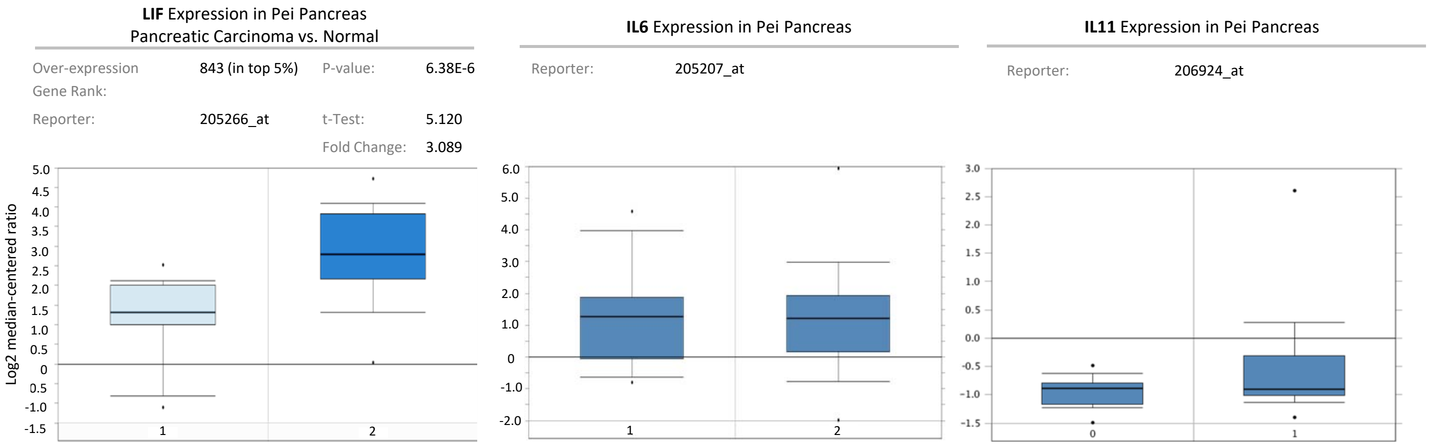

Pei Pancreas

Cancer Cell 2009/09/08 — 52 samples — 1. Pancreas (16) 2. Pancreatic Carcinoma (36)

Human Genome U133 Plus 2.0 Array — 19,574 measured genes

Supplementary Fig 1. LIF, but not other members in IL6 family, is regulated by KRAS.

(a) KRAS knock-down by shRNAs did not affect the expression of most IL-6 cytokine family members in human pancreatic cancer cell lines. qPCR was used to determine the expression level. β-actin was used as internal control, and expression level was normalized by control cell lines (N=3, *** $P < 0.001$; ** $P < 0.01$; * $P < 0.05$). Error bar represents the standard deviation and P value was generated by t test.

(b) Representative western blots showing the increase of phosphorylated STAT3 at Y705 in Panc1.0 cells where KRAS has been knocked down by different shRNAs.

(c) Oncomine analysis suggesting that IL-6, LIF and IL-11 mRNA expression in human pancreatic cancer and normal pancreas tissue.

Supplementary Figure 2

a

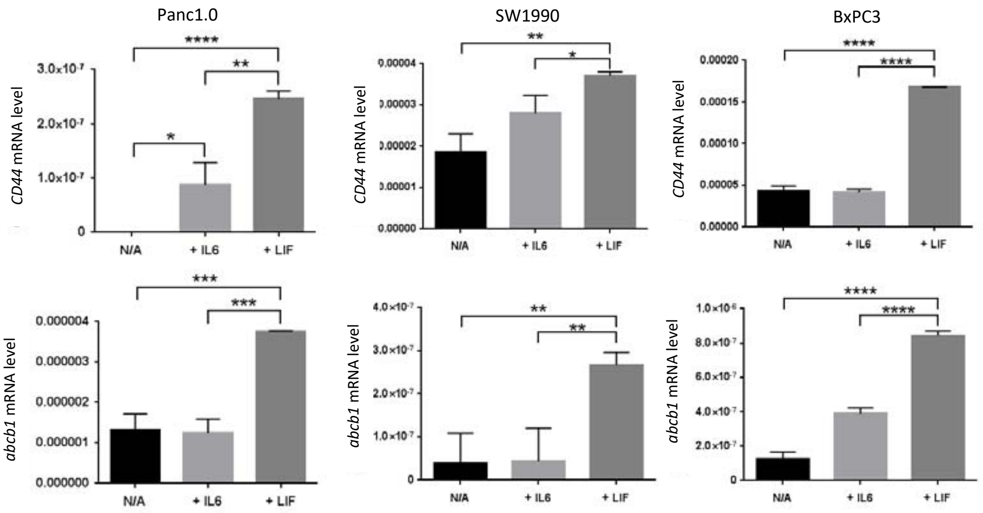

b

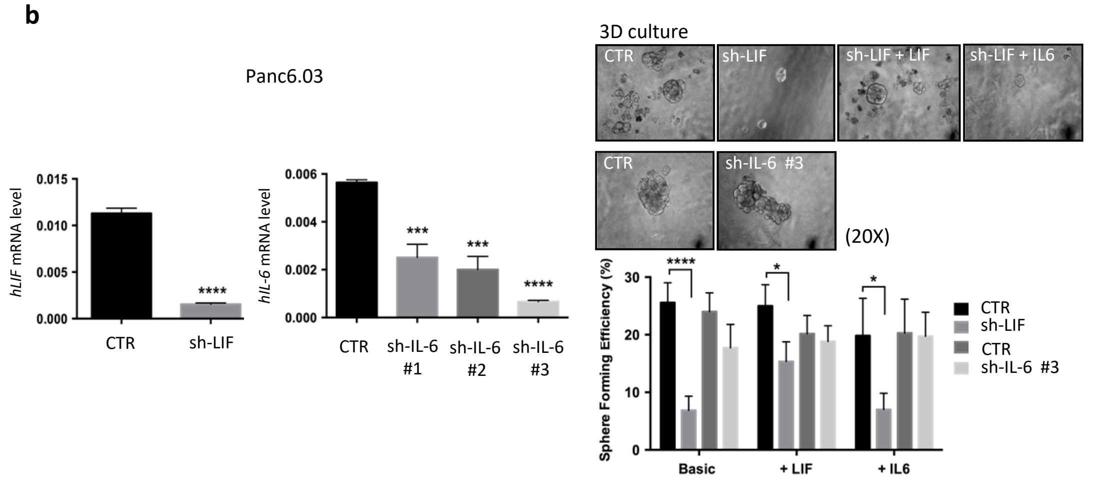

subcutaneous xenografts

Day 20th	0.1 X 10^6	1 X 10^6
CTL	5/5	5/5
sh-LIF	1/5	3/5
CTL	5/5	5/5
sh-IL-6 #3	4/5	5/5

c

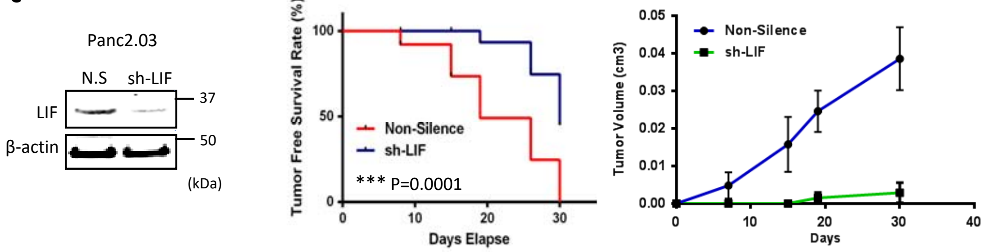

d

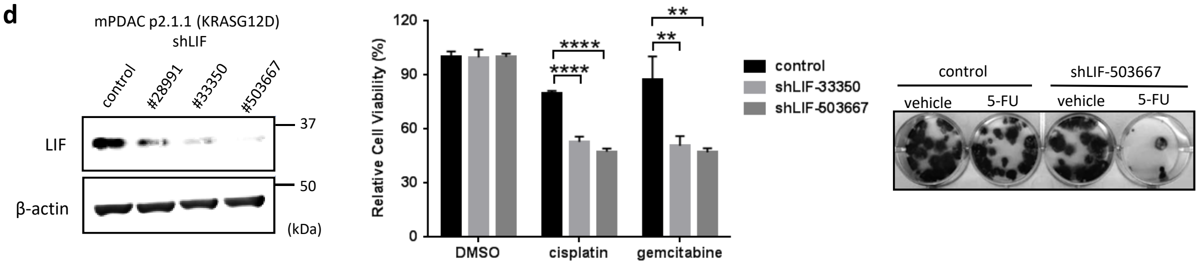

e

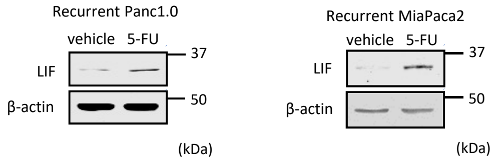

Supplementary Fig 2. LIF plays an essential role in mediating pancreatic cancer stem cell-like properties.

(a) Representative qPCR analysis showing mRNA expression of stem cell markers, *CD44* and *abcb1*, in different human pancreatic cancer cell lines treated with IL-6 or LIF at 100 ng/mL for 72 hours (N=3, * P < 0.05, ** P < 0.01, *** P < 0.001, **** P < 0.0001).

(b) (Left panel) qPCR showing mRNA expression of LIF or IL6 in Panc6.03 which express shRNAs against human LIF or IL-6 (N=3, *** P < 0.001, **** P < 0.0001). (Middle panel) Morphologies of spheres in Panc6.03 where IL-6 or LIF is knocked down. (Right panel) The number of xenograft tumors derived from Panc6.03 where IL-6 or LIF had been knocked down by shRNA.

(c) (Left panel) Western blots showing LIF expression in Panc2.03 which express shRNA targeting LIF. (Middle and right panel) Tumor free survival rate and growth curve of Panc2.03 xenograft tumors which express shRNA targeting human LIF (N=5).

(d) (Left panel) Western blots showing protein expression of LIF in mouse pancreatic cancer cells expressing different shRNAs targeting mouse LIF. (Middle panel) Cell viability in the same cell lines treated with cisplatin at 10μM or gemcitabine at 1μM for 72 hours. CellTiter Glo assay was used to determine the cell viability (N=6, ** P < 0.01, *** P < 0.001, **** P < 0.0001). (Right panel) Crystal violet staining showing re-growth of mouse pancreatic cancer cells expressing shRNA against LIF after the treatment of 5-FU at 50μM for 72 hours. Error bars (a)-(d) represent the standard deviation and P value was generated by t test.

(e) Representative western blots showing LIF protein expression in re-growing human pancreatic cancer cells after 5-FU treatment at at 50μM for 72 hours.

Supplementary Figure 3

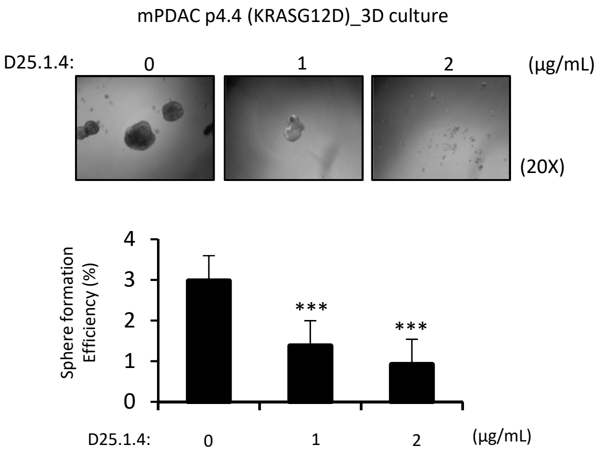

Supplementary Fig 3. Neutralizing LIF by antibody represses sphere forming ability of oncogenic KRAS-driven mouse pancreatic cancer cells.

Sphere formation assay in mouse pancreatic cancer cells treated with LIF antibody (N=6, *** $P < 0.001$). Error bar represents the standard deviation and P value was generated by t test.

Supplementary Figure 4

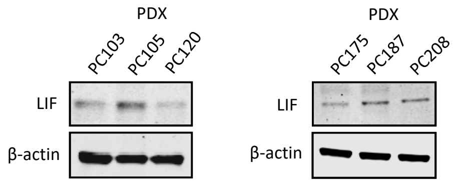

Supplementary Fig 4. Multiple PDX tumors express different protein levels of LIF.

Representative western blot showing LIF protein expression in different patient-derived xenograft (PDX) tumors.

Supplementary Figure 5

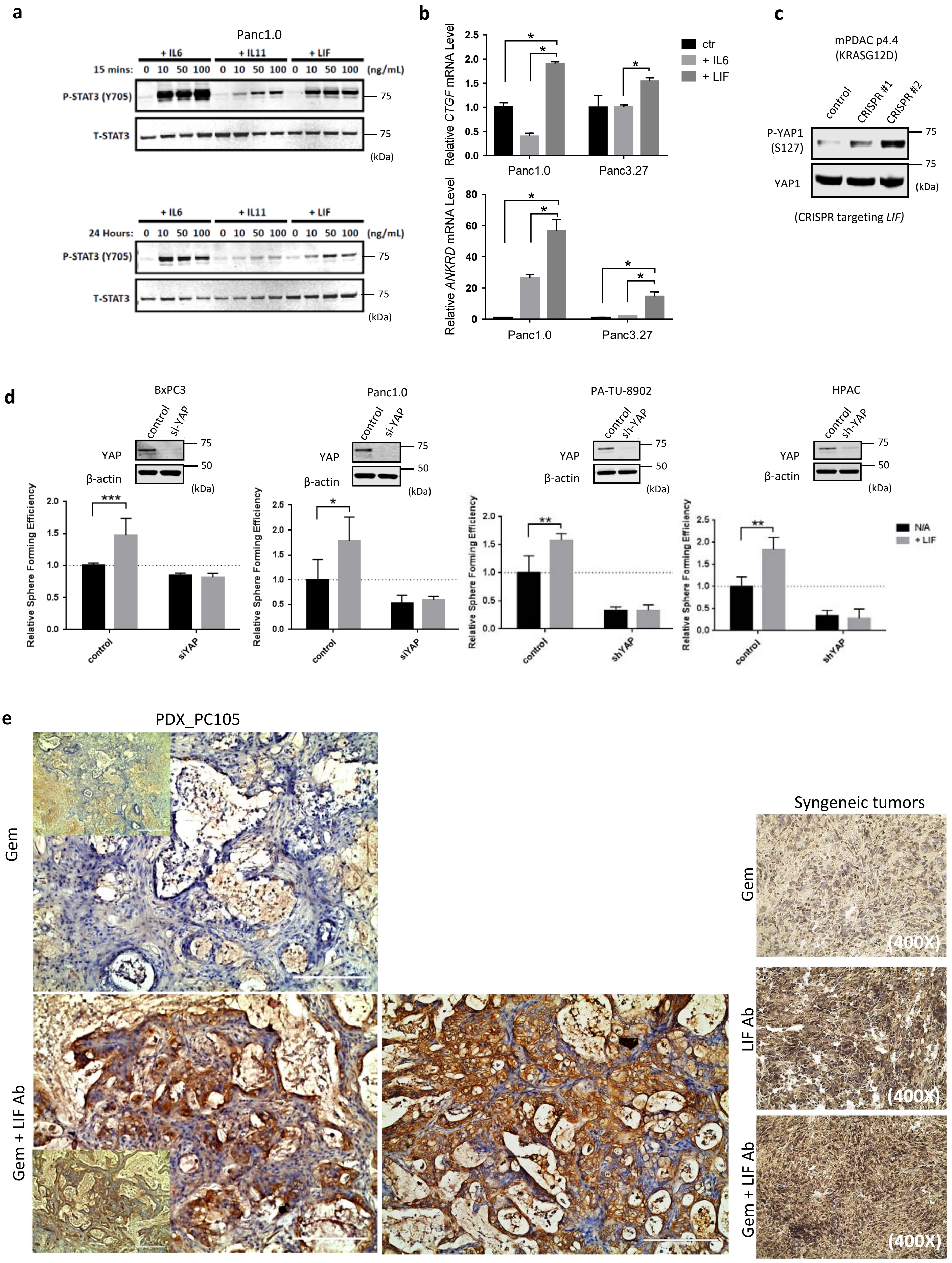

Supplementary Fig 5.

(a) Representative western blots suggesting that treatment with IL-6, IL-11, or LIF increased activation/phosphorylation of STAT3 in PANC1.0 cell line, but IL-6 showed better ability at the same concentration.

(b) qPCR showing mRNA expression of YAP targeted genes, *CTGF* or *ANKRD*, in Panc1.0 and PANC3.27 which were treated with IL-6 or LIF at 100ng/mL for 72 hours (N=3, * $P < 0.05$). Error bar represents the standard deviation and P value was generated by t test.

(c) Representative western blots showing that knocking out LIF by CRISPR/Cas9 increased phosphorylation of YAP at S127 in mouse pancreatic cancer cells. Total YAP was used as loading control.

(d) Sphere forming efficiency in multiple human pancreatic cancer cell lines where YAP is stably knocked down by shRNA in the presence or absence of human LIF in culture medium (N=6, * $P < 0.05$, ** $P < 0.01$, *** $P < 0.001$). Error bar represents the standard deviation and P value was generated by t test. Western blots in the upper panel showing YAP expression in different cell lines.

(e) (Left panel) Immunohistochemical staining for phosphorylated YAP at Ser127 in PDX tumors treated with gemcitabine and/or anti-LIF antibody. (Right panel) Immunohistochemical staining for phosphorylated YAP at Ser127 in syngeneic mouse pancreatic tumors with gemcitabine, anti-LIF antibody, or combination of gemcitabine and anti-LIF antibody.

Supplementary Table 1

Gene Name		oligo_sequences (5'-3')
hIL-6	Forward	ACTCACCTCTTCAGAACGAATTG
	Reverse	GTCGAGGATGTACCGAATTTGT
hIL-6	Forward	AATTCGGTACATCCTCGACGG
	Reverse	TTGGAAGGTTCAGGTTGTTTTCT
hCNTF	Forward	ACAGAGCATTCACCGCTGAC
	Reverse	TCAGGTCTGAACGAATCTTCCTT
hCNTF	Forward	GAAGATTCGTTCAGACCTGACTG
	Reverse	CAGGCCCTGATGCTTCACATA
hKras4B	Forward	ACAGAGAGTGGAGGATGCTTT
	Reverse	TTTCACACAGCCAGGAGTCTT
mKras4B	Forward	GAGTAAAGGACTCTGAAGATGTGCC
	Reverse	CATCGTCAACACCCTGTCTTGTCTT
hAbcb1	Forward	AGGAAGACATGACCAGGTATGC
	Reverse	CCAGCACCAATTCCACTGTAAT
hLIF	Forward	CAGTGCCAATGCCCTCTTTAT
	Reverse	GGCCACATAGCTTGTCCAGG
hLIF	Forward	CCAACGTGACGGACTTCCC
	Reverse	TACACGACTATGCGGTACAGC
hLIF	Forward	GTACCGCATAGTCGTGTACCT
	Reverse	GGTTGAGGATCTTCTGGTCCC
mLIF	Forward	AACCAGATCAAGAATCAACTGGC
	Reverse	TGTTAGGCGCACATAGCTTTT
mLIF	Forward	AGCTATGTGCGCCTAACATGA
	Reverse	CGACCATCCGATACAGCTCC
hCTGF	Forward	CAGCATGGACGTTCGTCTG
	Reverse	AACCACGGTTTGGTCCTTGG
hANKRD1	Forward	CGTGGAGGAAACCTGGATGTT
	Reverse	GTGCTGAGCAACTTATCTCGG
hCD44	Forward	GCCCTTCCATAGCCTAATCC
	Reverse	CTTTGGTGTCTCCCAGAAGC
hOSM	Forward	CACAGACTGGCCGACTTAGAG
	Reverse	AGTCCTCGATGTTCAGCCCA
hLeptin	Forward	TGCCTTCCAGAAACGTGATCC
	Reverse	CTCTGTGGAGTAGCCTGAAGC
hCLCF1	Forward	TTTCAACGAGCCAGACTTCAAC
	Reverse	GAGGCCACGCAAGTAACACA
hIL31	Forward	CACGTTGCCCGTCCGTTTA
	Reverse	TCTTCGAGAGGGACTGTAATTCC
hCT1	Forward	GGAGGCCAAGATCCGTCAG
	Reverse	AGCTGCACATATTCCTGGAGC
BACT	Forward	CAAGATCAACCGGGAAAAGATGA
	Reverse	TGGATGGCGACATACATGGC

Supplementary table 1. The list of oligos used in qPCR assays
