# Supplementary figures and images for: Blockade of leukemia inhibitory factor as a therapeutic approach to KRAS driven pancreatic cancer
Source: Nat Commun. 2019 Jul 11;10:3055. doi: 10.1038/s41467-019-11044-9 (PMC6624260; doi:10.1038/s41467-019-11044-9)

Fig 2a.

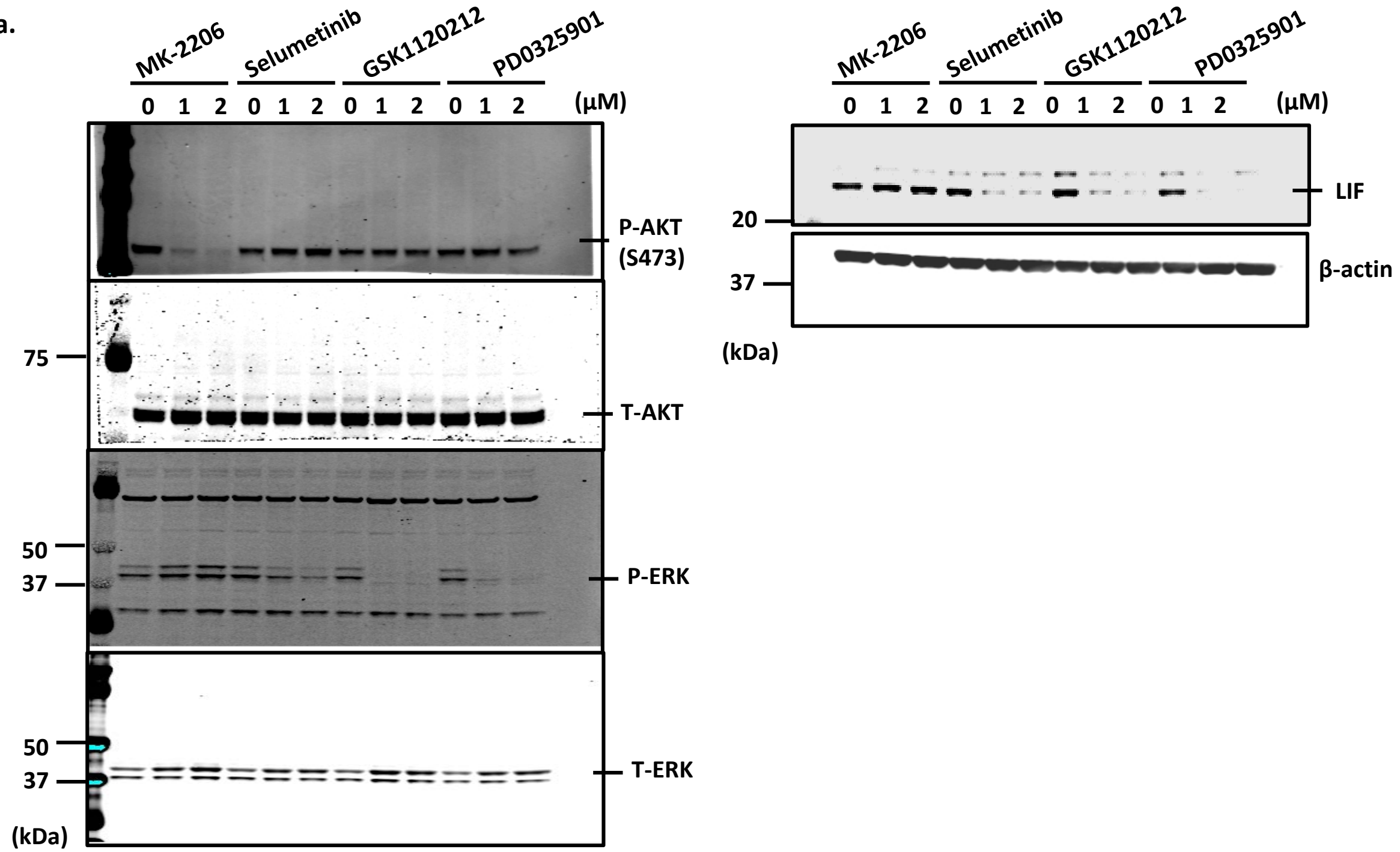

Fig 4b.

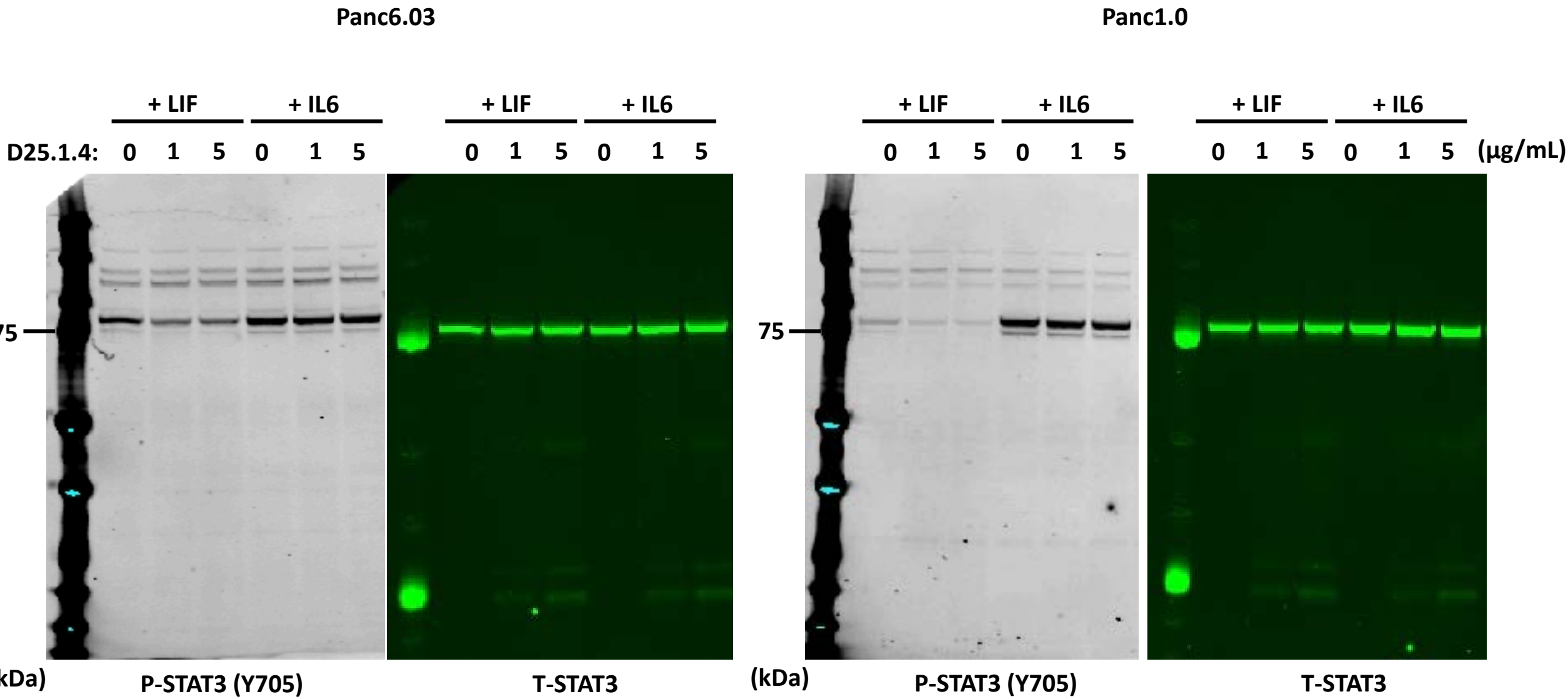

Fig 5b.

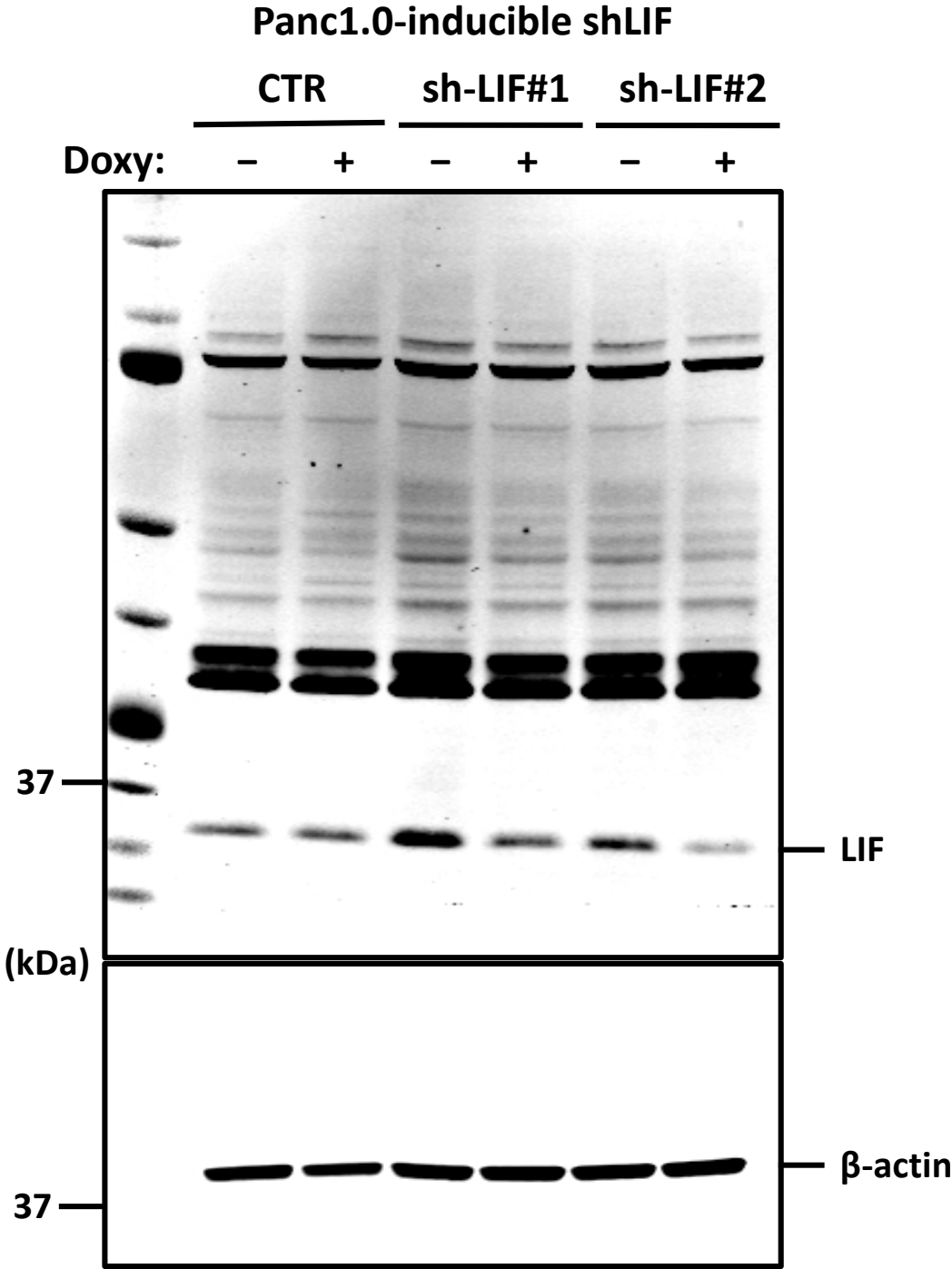

Fig 5d.

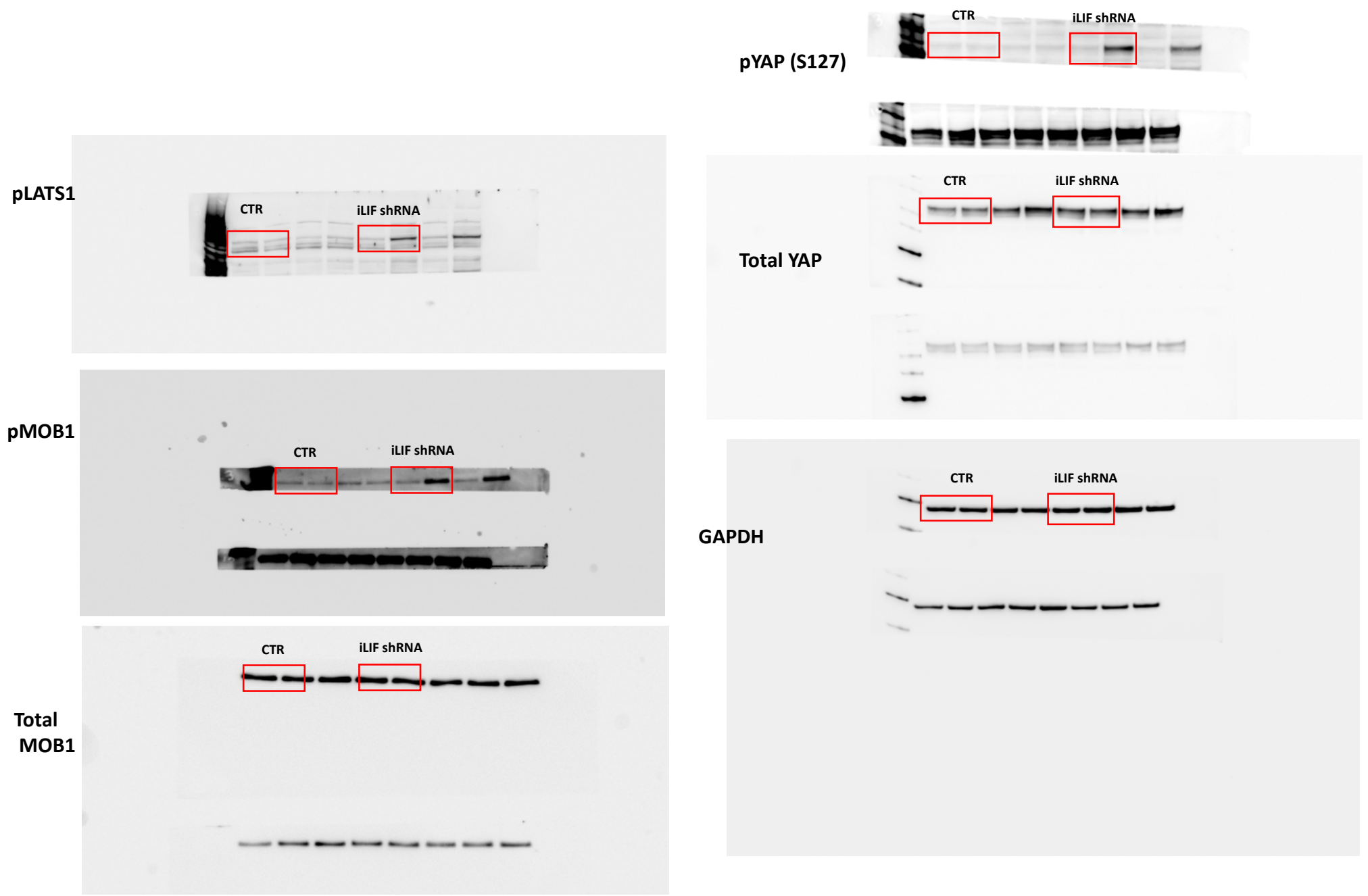

Fig 5e.

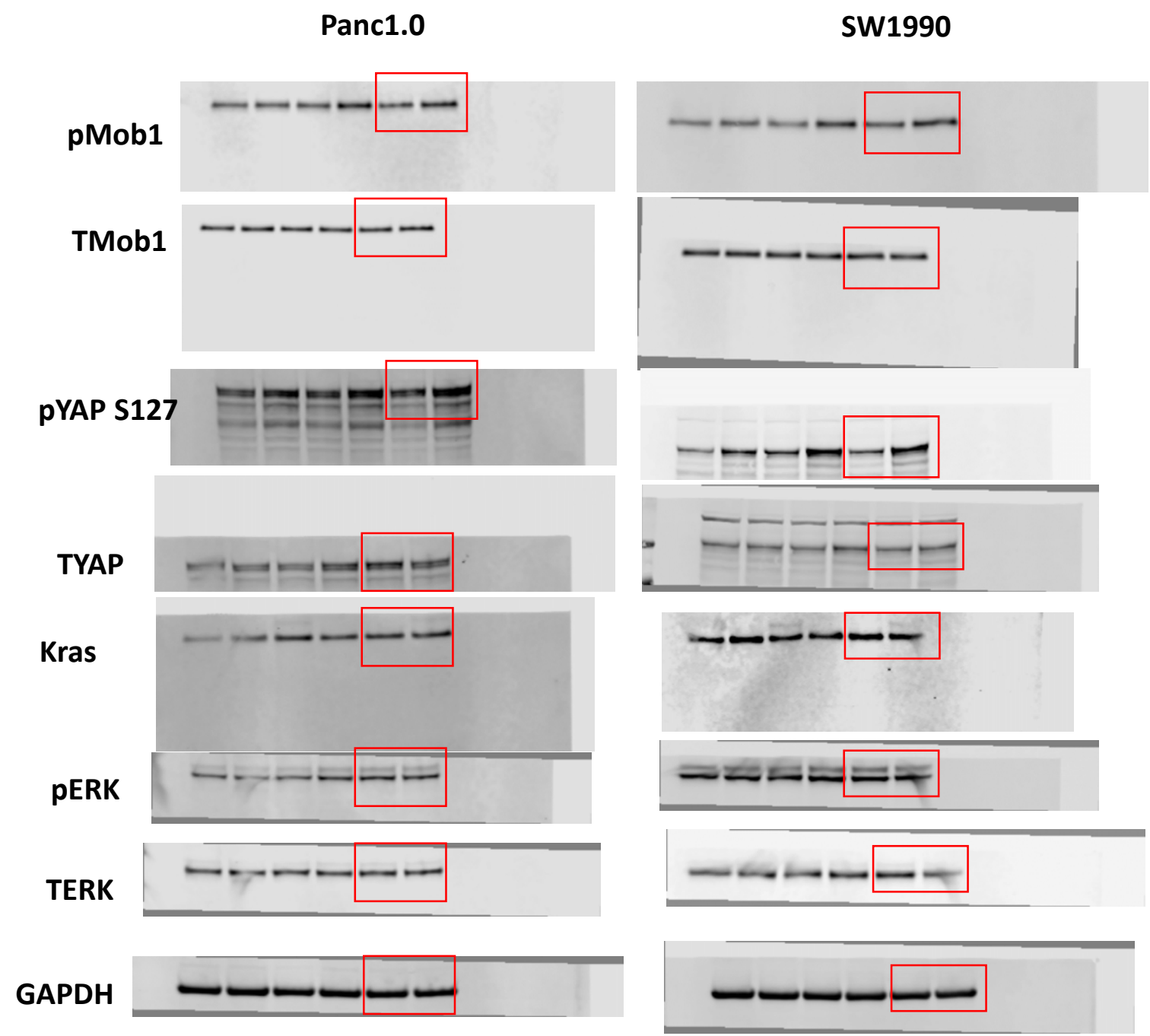

Supplement: Supplementary file 5 — Source Data [file 41467_2019_11044_MOESM5_ESM.pdf]
